# Commentary on “Single-cell transcriptomic analysis reveals therapeutic mechanisms of adipose-derived stem cell exosomes in sepsis-induced lung injury”

**DOI:** 10.1097/JS9.0000000000005151

**Published:** 2026-03-18

**Authors:** Yue Liu, Yuchao Zhou, Wendi Liu, Tao Sun

**Affiliations:** a Dalian Medical University, Dalian, Liaoning, China; b Department of Critical Care Medicine, The Second Hospital of Dalian Medical University, Dalian, Liaoning, China; c Department of Infectious Disease, The Second Hospital of Dalian Medical University, Dalian, Liaoning, China

*Dear Editor:*

We read with great interest the article by Wu *et al*[1] exploring the immunomodulatory effects of adipose-derived stem cell exosomes (ADSC-Exos) in a murine model of sepsis-induced lung injury using single-cell RNA sequencing. By integrating transcriptomic profiling with experimental validation, the authors provide novel insights into immune-cell heterogeneity and altered intercellular communication during septic lung injury. While the study is innovative, several issues merit discussion to improve translational interpretability. This commentary adheres to the Transparency In The reporting of Artificial INtelligence (TITAN) guideline[2].

First, the study relies exclusively on a murine cecal ligation and puncture (CLP) model, which only partially reflects the complexity of human sepsis. Marked differences between murine and human immune responses have been documented, particularly in leukocyte activation patterns and temporal immune dynamics, limiting direct clinical extrapolation of transcriptomic findings[3].

Second, although single-cell RNA sequencing enables high-resolution characterization of cellular states, validation of the reported cellular states and trajectories would be strengthened by the use of independent cohorts or complementary techniques. Orthogonal approaches such as flow cytometry or spatial transcriptomics could help corroborate key immune-cell phenotypes identified by single-cell RNA sequencing, thereby enhancing robustness and reproducibility[4]. Independent validation using complementary approaches would strengthen confidence in the reported findings.

Third, the therapeutic timing and dosing strategy of ADSC-Exos warrant further clarification. In clinical sepsis, therapeutic windows are often heterogeneous and unpredictable, whereas preclinical studies typically administer interventions at fixed time points following CLP induction. Prior translational studies have demonstrated that the timing of immunomodulatory therapies critically influences sepsis outcomes, with early and delayed administration yielding divergent effects[5]. Evaluation of alternative dosing strategies or delayed treatment paradigms may therefore enhance clinical relevance.

Moreover, although attenuation of inflammatory signaling pathways and restoration of intercellular communication networks are reported, the durability of these transcriptomic changes remains uncertain. Sepsis-induced lung injury frequently evolves through phases of hyperinflammation, immunosuppression, and tissue remodeling, and short-term molecular improvements do not necessarily translate into sustained functional recovery or survival benefit[6]. Longer-term outcome assessments would provide important context for interpreting the biological significance of these findings.

Finally, despite increasing recognition of the immunomodulatory potential of exosome-based therapies, challenges related to exosome heterogeneity, cargo variability, and methodological standardization persist. Variations in isolation, characterization, and storage protocols can substantially influence biological activity, thereby limiting reproducibility and clinical translation[7]. Greater methodological transparency and standardization will be essential as this therapeutic strategy advances toward clinical application.

In conclusion, Wu *et al* present a valuable single-cell transcriptomic analysis elucidating potential mechanisms by which ADSC-Exos modulate immune responses in sepsis-induced lung injury. Addressing limitations related to animal modeling, sample size, therapeutic timing, and translational applicability will be essential to support future clinical development of this promising therapeutic approach.

## Data Availability

The data can be obtained from the corresponding author upon reasonable request.
